# Person-centred healthcare and medicine paradigm: it’s time to clarify

**DOI:** 10.1186/s13167-015-0033-3

**Published:** 2015-05-16

**Authors:** Paolo Roberti di Sarsina, Mariateresa Tassinari

**Affiliations:** Charity for Person Centered Medicine-Moral Entity, Bologna, Italy; Observatory and Methods for Health, University of Milano-Bicocca, Milan, Italy

**Keywords:** Person-centred healthcare and medicine paradigm, Traditional Complementary and Alternative Medicine, Health policy

## Abstract

The person-centred healthcare and medicine paradigm is in need of a strong theoretical framework not only to explain what it is but to prevent dangerous confusions of terminology or reductive oversimplification of its true scope: for example, it may be integrated into biomedicine, whereas person-centred medicine and Traditional Systems and Complementary and Alternative Medicine (TCAM) actually stand in the position of interacting with the conventional health system. Emphasis on person-centred care is also in line with World Health Organization (WHO) policy and the International Declarations of Beijing and Alma Ata. Interaction of TCAM and person-centred approach to all forms of medicine will ensure variety of therapy in tackling the intrinsically complex and multifaceted issue of health and healing. It will also prevent inestimable traditional knowledge from being lost.

## Review

### Introduction

A paradigm is a reference model by definition, a set of rules and explanations giving it a benchmark quality. Its main feature is that the terms by which it manifests confer upon it a precisely denoted meaning. While such linguistic definition suffers from the intrinsic disadvantage of hedging the paradigm round in epistemology, it does enable it to fulfil both a prescriptive and a descriptive function. It thus becomes clear that we cannot proceed to switch terminology (‘patient’ for ‘person’ would be an example of the case in point), which indirectly offsets the idea that practice has superior standing in terms of value. No: medicine is made up of actions *but also theories* [1], people not only act but argue and reach agreements. All of which makes it necessary to clarify just what the person-centred healthcare and medicine paradigm exactly means [2], and why it is the only course open to sustainable medicine in the twenty-first century.

We use the term person and not patient so as not to distort our paradigm. It does not just amount to an attitude of politeness and empathy giving the patient due attention: that is deontology, nothing more. Nor does the issue boil down, as is commonly thought, to rising above the Cartesian framework of body-mind and embracing a holistic view of the person, instead of considering him a bundle of organs. To our way of thinking, that is simply the basic premise from which we must set out; it is not an achievement on which to preen ourselves [3]. To define person-centred medicine in such terms is to demean and reduce the paradigm, cramping its potential.

### What is it necessary to clarify?

What we perceive as an urgent need for clarification is the ontological and epistemological framework of the paradigm in question and its practical implications. In fact, often, there’s a tendency to rather superficially include all medical approaches in which the patient plays a fundamental importance in the great macro category of Medical Humanities. In order to avoid misunderstanding, we want to assign a specific identity rather than give value to another. For example, the person-centred medicine and medical paradigm is not only prevention but salutogenesis not prediction through costly genetic testing that could (potentially) contribute to an increased inequality in terms of health but also empowerment to the citizen.

We will now go on to explain the peculiarities.

### What does ‘person’ at the centre mean?

Even before the patient is identified as such, medicine needs to be person centred in real and not just formal recognition of the dignity of every sentient being. This will enable the suffering person to decide for herself (or himself) and will give due attention to her/his beliefs, creed, spirituality and the culture to which she belongs and her personal sensibilities. Person-centred medicine goes even further. It is a way of reducing health inequality; it empowers the person and keeps treatment sustainable. It is health-generating (salutogenesis) and not just prevention [4].

**Figure 1:**
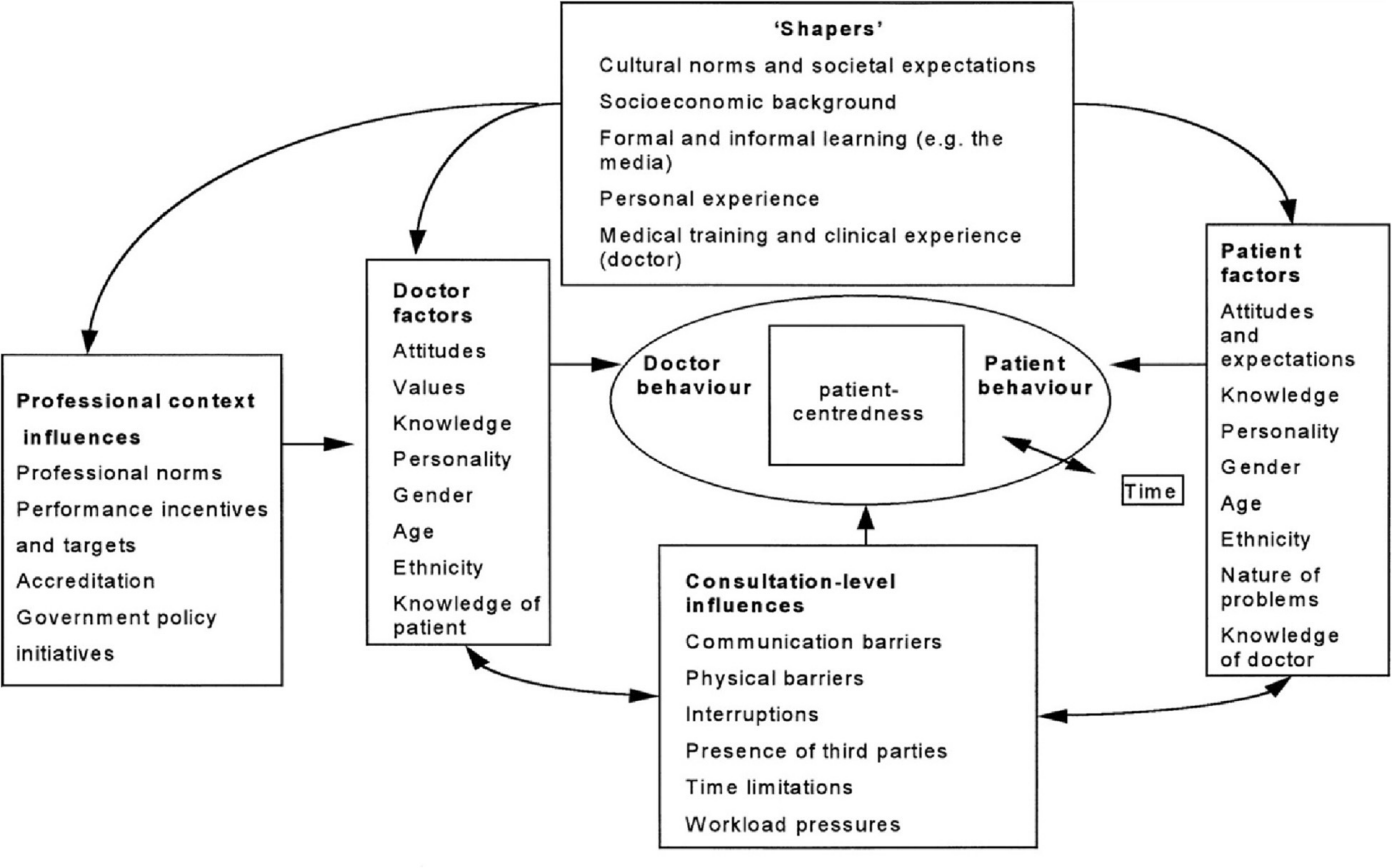
Factors affecting patient-centredness.

Health and medicine are only properly centre on the person [5,6] if that person is involved from the outset, if she is able to work towards a plausible course of action and an acceptable form of treatment, if she/he is really enabled to take part in decision-making over what therapy to adopt, if her/his religion and spirituality are consulted in the process of healing, and if *all the determinants of health are duly weighed* (Figure 1) [7]. The Declaration of Geneva, 2014, confirms that this must necessarily be so [8].

### Why is the role covered by TCAM crucial in the person-centred medicine and healthcare paradigm?

As in Traditional Systems and Complementary and Alternative Medicine (TCAM), person-centred medicine is a way of treating and caring for the whole person: assessing diet, safeguarding the environment, removing obstacles that might block or curtail health-enhancing practices [9,10] and, lastly, focusing on social relations and all other determinants of health [11]. As it is well known, an international consensus from the semantic point of view is still missing. *Complementary and Alternative Medicine* (CAM) is a widely used term, but it does not have a commonly accepted definition. It was developed at a 1997 conference of the United States Office for Alternative Medicine of the National Institutes of Health (now National Center for Complementary and Alternative Medicine (NCCAM)) and subsequently adopted by the Cochrane Collaboration and the Ministerial Advisory Committee on Complementary and Alternative Medicine. At the same time, the World Health Organization in ‘General Guidelines for Methodologies on Research and Evaluation of Traditional Medicine’ (Geneva, 2000) claims ‘Traditional Medicine’ as being in deference to the nations and cultures where such forms of medicine are an integral part of the cultural and medical heritage (for instance, China’s and India’s cultural traditions). Traditional Medicine is the total sum of indigenous knowledge used in the maintenance of health in these countries; with the aim of being scientifically neutral, we decided to unify in a unique acronym CAM and Traditional Medicine.

To foster health by a pro-active, pro-resilient health-generating approach marks a point of historic crux and transition. From being an *object* of observation and welfare (to be ‘patched up’ and restored to his/her place of provenance as quickly as possible), the patient becomes an active and responsible *subject*, the true protagonist in the process of healing.

Health is truly a matter for individual responsibility [12], but above all it is a right that needs preserving by governments and is not just a ‘good’ that should be entrusted to Medicine. The challenges facing present-day society emphasise this point.

A clear gap can be discerned between the notions of ‘health care’ and ‘health system’—a gap to be reckoned with as we strive to re-programme health in the light of each country’s mounting economic difficulties. The health system by no means covers all the aspects of health; we need to ponder *all* the significant features of a health system and link up the patient’s physical symptoms with all planes of her or his existence.

TCAM is based on the premise that wellbeing is intrinsically and ontologically bound up with the whole person, and that the individual must be seen as an indivisible union of body, mind and spirit, all of whose behavioural, psychological, spiritual, environmental and cultural features need to be understood [13,14].

### Is what we call placebo the ‘person-centred medicine’?

In our opinion, this argument becomes abundantly clear when we ask ourselves why placebos work [15]. In support of this position, we will briefly examine the case of placebos in acupuncture. We should first decide upon the conditions enabling us to talk about placebo *ontology* or *epistemology*: that is, can one study the placebo phenomenon as an entity in itself, or is it more correct to speak of the relationship between the subject taking cognizance and the object being studied?

Such a cognitive issue hinges on the recognition that it is reductive on a micro level, to distinguish *à la* Descartes between mind and body, and above all on a macro level, to fail to take into account the environment, beliefs and culture attaching to every individual person. If we eliminate the dualism, we are forced to ask: what remains of the placebo? In this regard, we need to understand if the efficacy of a placebo is always one and the same or if we need to distinguish the effect of swallowing a tablet of sugar from having a needle stuck into us. Pace the common view, sham acupuncture is not really inert: as we shall see, a needle penetrating the skin is more effective than placebo pills.

It is hard to find a clear-cut answer to the problem posed above. Many factors are involved [16] such as the diagnosis of disease, the state of health of those taking part in the study, the research model, etc. It is easy to see how studies designed to answer the question tend to come up with different results.

In 2010, a Cochrane meta-analysis [17] was performed on placebo effects and the possible differences in their effectiveness. It was found that 61 trials with ‘physical’ placebos proved more effective than response to a pharmacologically inert pill. The results of that study were re-analysed and the evidence was this time found to be highly heterogeneous in terms of patients, method of intervention and results [18]; but nonetheless, the researchers did suggest that sham acupuncture tends, on average, to have a greater effect than a physical placebo. Another study [19], though not a review or a meta-analysis, serves as a useful crosscheck for assessing the possible lack of uniformity in the participants’ response to placebo pills, sham acupuncture, real acupuncture or control status. One key question posed by that study was whether the response to placebo could be affected by any particular circumstances.

The researchers found no difference between the effects of sham acupuncture and an inert pill when it came to measuring the threshold of pain, tolerance of pain or appraisal of pain. They also found that response to real acupuncture positively correlated with response to sham acupuncture. This supports the view that the non-specific effects of acupuncture play a significant part in the end results of real acupuncture. For the moment, though, we should focus on the problems emerging from the two articles we have mentioned.

One of the chief differences between the two studies is the nature of what was being observed: in the first case, the people were suffering from a disease; in the second, they were healthy. This means that what varies is not just the perception or tolerance of pain but concerns the mechanisms by which we realise that a painful experience is or is not to do with illness. An artificially induced pain is hardly the same as one felt when suffering from an illness. The physical suffering of disease is only a fragment of the social and emotional effects caused by being ill. Besides, the pain inflicted during the experiment was short-lived, while the people observed in the Cochrane meta-analysis did not have anything like the same certainty.

More food for thought comes from the differing effects achieved in the first study by a ‘physical’, as opposed to a pharmacological, placebo. A healing pill is a socio-cultural symbol, swallowing it a stepping stone to cure. Evocative though that power is, the study nonetheless shows that the placebo effect is stronger when a physical, not a pharmacological, placebo is being used, calling for a health worker to administer it.

This fact is crucial not only for placebos but above all for therapy itself. It also goes to show how fragile a name is to describe an idea.

To put the words or presence of a health worker on a par with a placebo is far from indifferent: we consider it to be based on an ill-founded pre-conception whereby the centrality of the person in the treatment/healing process is lost from sight. In the case of acupuncture, or TCAM in general, patients are unconsciously more exposed sets of variables. This may be connected with the popular idea of traditional health systems or it may be due to a time factor: the time an expert practitioner of such medicines devotes to a physical examination and talking to the patient, all of which builds up confidence. Patients have expectations, they ask friends or relatives for advice, they read magazines and surf the web.

All kinds of treatment, whether conventional or not, engender two types of effect, specific and non-specific: it is an inseparable synergy of these [20] that creates the therapeutic potential [21].

If the patient and the doctor’s expectations of a treatment can bear markedly on the outcome, if the setting in which that treatment takes place produces a pronounced effect on the patient’s emotional state, that is surely the starting point from which we can understand how important and cost-effective it is to put the person in the centre of the therapy process [22,23].

### Practical implication

If we analyse the TCAM issue from the angle of international policy, we should recall that in the Alma Ata Declaration on Primary Health Care [24] and in the Traditional Medicine strategy re-launch 2014–2023 [25], the World Health Organization (WHO) appealed to the international community to support inclusion of TCAM (when scientifically shown to be effective) in national health systems. The grounds for this are that TCAM can reduce the consumption of conventional drugs and the cost of public health systems: ‘Health systems around the world are experiencing increased levels of chronic illness and escalating health care costs. Patients and health care providers alike are demanding that health care services be revitalised, with a stronger emphasis on individualised, person-centred care’ [26].

Applying a person-centred healthcare and medical paradigm, as with TCAM, reduces adverse patient reaction to drugs [27]—a major issue of conventional drug treatment (which hence becomes a double health hazard if one remembers their strongly *pollutant* nature) [28]. Manufacturing and using TCAM products also boosts the local economy and helps to make local health services sustainable. It preserves forms of knowledge that have developed in various ways and situations, and thus leads to a multicultural, multidisciplinary and pluralistic approach to the question of health. A multi-sectorial approach must be adopted so as to include the role of the social determinants of health while, as mentioned, government-funded research and development needs to address these challenges. This strategy was advocated in the Beijing Declaration [29].

In rich and poor countries alike, it is important that the interaction between traditional knowledge and conventional medicine be taught at university so that students can learn how health practices have evolved in our various countries [30,31]. This will also protect people from unethical or misguided practices, and thus extend the good standards achieved by biomedicine. Lastly, the traditional health systems provide an important way of incrementing the capacity of public health systems to improve people’s quality of life: ‘Although there are common themes underlying the reasons which motivate people to use TCAM, there are also many differences between individual countries and regions. Some studies have shown that individuals choose TCAM for various reasons, including an increased demand for all health services, a desire for more information leading to an increased awareness of available options, an increasing dissatisfaction with existing healthcare services, and a rekindled interest in ‘whole person care’ and disease prevention which are more often associated with TCAM. In addition, TCAM recognises the need to focus on quality of life when a cure is not possible [32]’.

## Conclusions

Why then are TCAM so important in health care and person-centred medicine?

There are many reasons—since these are natural methods of healing, treating the person rather than the symptoms, or boosting the patient’s innate self-healing ability, and so on; but if we had to choose *one* reason, we think the best answer is that these health systems are the proof that person-centred medicine is not just necessary but above all feasible [33-35]. The conditions for making it so may be summed up in a nice distinction of terms *interaction* (between TCAM and Biomedicine) not integration:

- No single hierarchical system can claim to interpret the complex whole;
- We must ensure such traditional knowledge survives [36,37]; and
- Such diverse systems of acquired knowledge [38] need faithfully preserving and translating, (to translate not to betray) on the understanding that different backgrounds and ways of living call for specific treatment tailored to the circumstances.

Only by observing these three points can we hope to transform theory—of the kind we have just been relaying—into practice. The current—and in our opinion misguided—paradigm that sees the interaction of biomedicine with TCAM as ‘Integrated Medicine’ will in this way be corrected.

### Recommendations for experts

In light of has been stated, experts involved should:

- Not consider the body within a solely biological context but also rituals, religious and historical;
- Give the same importance to all stages and all the stakeholders of the disease;
- Understand that the ‘end result’ according to the doctor does not always coincide with that of the patient; and
- Educate patients in order to permit them to become consciously active in their disease, respect the wishes and needs of women from other cultures, to which it is a human necessity to ensure that their needs are satisfied, reconsider how places of care are designed, and teach stress management.
